# Blockade of miR-140-3p prevents functional deterioration in afterload-enhanced engineered heart tissue

**DOI:** 10.1038/s41598-019-46818-0

**Published:** 2019-08-07

**Authors:** Tessa R. Werner, Ann-Cathrin Kunze, Justus Stenzig, Thomas Eschenhagen, Marc N. Hirt

**Affiliations:** 1 Department of Experimental Pharmacology and Toxicology, University Medical Center Hamburg-Eppendorf, Hamburg, Germany; 2 DZHK (German Centre for Cardiovascular Research), partner site Hamburg/Kiel/Lübeck, Hamburg, Germany

**Keywords:** Tissue engineering, miRNAs, Cardiac hypertrophy

## Abstract

Afterload enhancement (AE) of rat engineered heart tissue (EHT) *in vitro* leads to a multitude of changes that *in vivo* are referred to as pathological cardiac hypertrophy: e.g., cardiomyocyte hypertrophy, contractile dysfunction, reactivation of fetal genes and fibrotic changes. Moreover AE induced the upregulation of 22 abundantly expressed microRNAs. Here, we aimed at evaluating the functional effect of inhibiting 7 promising microRNAs (miR-21-5p, miR-146b-5p, miR-31a-5p, miR-322-5p, miR-450a-5p, miR-140-3p and miR-132-3p) in a small-range screen. Singular transfection of locked nucleic acid (LNA)-based anti-miRs at 100 nM (before the one week AE-procedure) led to a powerful reduction of the targeted microRNAs. Pretreatment with anti-miR-146b-5p, anti-miR-322-5p or anti-miR-450a-5p did not alter the AE-induced contractile decline, while anti-miR-31a-5p-pretreatment even worsened it. Anti-miR-21-5p and anti-miR-132-3p partially attenuated the AE-effect, confirming previous reports. LNA-anti-miR against miR-140-3p, a microRNA recently identified as a prognostic biomarker of cardiovascular disease, also attenuated the AE-effect. To simplify future *in vitro* experiments and to create an inhibitor for *in vivo* applications, we designed shorter miR-140-3p-inhibitors and encountered variable efficiency. Only the inhibitor that effectively repressed miR-140-3p was also protective against the AE-induced contractile decline. In summary, in a small-range functional screen, miR-140-3p evolved as a possible new target for the attenuation of afterload-induced pathological cardiac hypertrophy.

## Introduction

A critical increase in the worldwide prevalence of heart failure in the next decades seems to be unavoidable. It is well known that aging of the population contributes significantly to this increase^1^. Less known is that among younger individuals (18–50 years) the incidence of cardiovascular disease increases due to obesity, physical inactivity and poor diet which will even aggravate the situation in the future^2^. In the last 30 years the mortality of patients suffering from chronic heart failure has decreased owing to the treatment with inhibitors of the renin-angiotensin-^3,4^-aldosterone-^5^-system, and beta-blockers^6^. While the first 15 years of this period brought up four prognostically favorable drug classes, the second half was characterized by 13 years (2001–2014) during which no new drug class showed benefits on mortality of heart failure patients^7^ and the focus shifted to a better implementation of existing heart failure guidelines in the clinical routine^8^. In 2014 however, the PARADIGM-HF-trial demonstrated that a new therapeutic principle, namely the increase of natriuretic peptides, is able to improve the prognosis of patients even when added to a complete guideline conforming heart failure medication^9^. This encouraging result further motivated research on other molecular principles that might inhibit heart failure progression, e.g. the emerging field of non-coding RNAs.

More than 95% of all transcribed human RNAs do not encode for proteins. The most promising therapeutic targets comprise three main subclasses of non-coding RNAs: microRNAs, long non-coding RNAs and circular RNAs^10^. In heart failure and one of its major risk factors, pathological cardiac hypertrophy, the best studied class is microRNAs^11,12^. This is due to several facts: MicroRNAs were the first regulatory non-coding RNAs to be discovered^13^. Moreover, in contrast to lncRNAs and circRNAs, microRNAs are highly conserved among species and their mature form (miRs) are relatively simple molecules consisting of 18–25 nucleotides. Thus, target identification and inhibition appears to be simple, namely by reverse complementary synthetic oligonucleotides. The effect of microRNA modulation on heart function is not necessarily mediated by regulation of gene expression in cardiomyocytes. Other cardiac cell types like fibroblasts, or endothelial cells can also be the primary target^14^. As such, the full functional effect of a microRNA modulation (often inhibition) can only be observed when all cardiac cell types are present in work performing cardiac tissue. Therefore, experiments in rodent hearts have been indispensable.

In recent years, we have been developing work performing engineered heart tissue (EHT) from rat hearts containing a mixture of all cardiac cell types^15^. An increase in afterload of these EHTs *in vitro* led to a multitude of changes that are known *in vivo* as pathological cardiac hypertrophy: cardiomyocyte hypertrophy, contractile dysfunction, impaired relaxation, reactivation of fetal genes (e.g., ANP or BNP), metabolic changes, necroptosis and fibrotic changes^16^. Moreover, hypertrophied EHTs show a distinct microRNA-signature with 22 abundantly expressed upregulated microRNAs and 15 abundantly expressed downregulated microRNAs^17^. In the current study, we evaluated the functional effect of inhibiting 7 promising microRNAs found to be upregulated in the previous study.

## Results

### Choice of chemistry to block microRNAs in EHTs efficiently

Two different chemistries of anti-miRs were tested. ‘Antagomirs’ are DNA-oligonucleotides, which are reverse complementary to the full-length microRNA and carry five phosphorothioates as well as a cholesterol-tag on the 3′-end. LNA-anti-miRs are also reverse complementary sequences but usually shorter than the target microRNA. Furthermore, approximately every third to fourth DNA-nucleotide is replaced by a locked nucleic acid (LNA) and all phosphodiesters are replaced by phosphorothioates, which ensures high endo- and exonuclease stability and high affinity binding to the target microRNA. Antagomirs turned out to be comparatively ineffective in the EHT-system. To achieve >80% reduction of any active target microRNA (miR-24-3p), an antagomir concentration of 80 µg/ml (9.88 µM) in the cell culture medium had to be applied (Fig. S1a). This concentration however, appeared to be acutely toxic for EHTs, as it led to a reduction in contractility of EHTs by more than 50% in 3 days, regardless of the targeted sequence (Fig. S1b). Although we did not directly compare antagomirs and LNA-anti-miRs targeting the same microRNA, the latter seemed to be much more potent, as e.g. a >98% reduction of active target microRNA (miR-21-5p) was provoked when EHTs were treated at an LNA-anti-miR-concentration of 20 µg/ml (4.4 µM) in the medium^17^. Moreover, LNA-anti-miR-EHTs showed no signs of acute or chronic toxicity^17^. In order to cut the cost-intensive LNA-anti-miR-consumption and thus facilitate a small-range screen, we also investigated other methods than unassisted delivery of LNA-anti-miRs at high concentrations. A cationic polymer mediated transfection at only 100 nM concentration for 2–3 days also led to a sufficient intracellular delivery of LNA-anti-miRs without any signs of toxicity and thus reduced the LNA-anti-miR-consumption and expenses by a factor of 44 (Figs S1c,d and S2).

### Choice of microRNAs to be blocked

In our previous study^17^ we had observed upregulation of 22 abundantly expressed microRNAs by the AE-procedure: miR-21-5p, miR-146b-5p, miR-208b-3p, miR-351-5p, miR-31-5p, miR-21-3p, miR-322-5p, miR-210-3p, miR-450a-5p, miR-322-3p, miR-140-3p, miR-182-5p, miR-221-3p, miR-542-3p, miR-214-3p, miR-222-3p, miR-34a-5p, miR-422a-5p, miR-1983-3p, miR-378i-5p, miR-210-5p, miR-132-3p (in descending order of sequenced reads). For our small-range screen we chose 7 microRNAs to be blocked (Table 1), all of which are conserved in humans with maximally one mismatch at the 3′-end and thus far away from the important seed sequence. Two very different anti-miRs (anti-miR-21-5p and anti-miR-132-3p) were intended as positive controls for the attenuation of the AE-induced contractile impairment: the inhibition of the highly expressed, fibroblast-enriched miR-21-5p, which has been reported to be pro-fibrotic^18^, and the inhibition of the lowly expressed, cardiomyocyte-enriched miR-132-3p, which has been reported to be pro-hypertrophic^19^. The inhibition of miR-146b-5p, miR-31a-5p, miR-322-5p and miR-450a-5p appeared to be promising as these microRNAs were highly expressed and strongly upregulated by AE, but not yet well characterized in the literature. Lastly, the inhibition of miR-140-3p was chosen, as miR-140-3p has been misclassified as minor (“star”) microRNA and therefore might have been overlooked on earlier conventional array based screens.

**Table 1:**
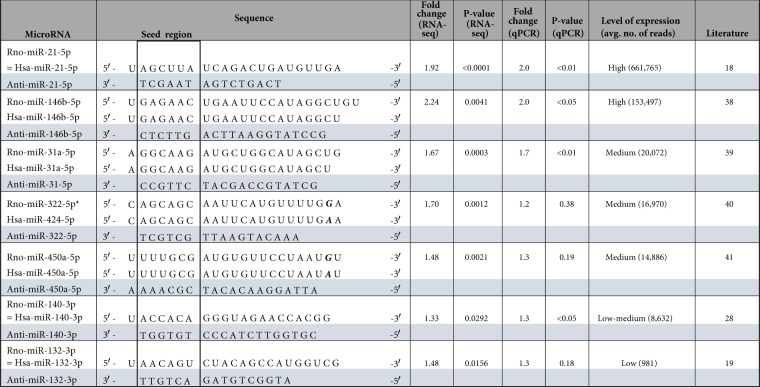
Overview of microRNA sequences and differential expression analysis in AE-EHTS compared to control EHTs.

*previously Rno-miR-424.

The regulation of all seven chosen microRNAs by AE was reevaluated by quantitative microRNA-PCR (Table 1) which numerically well confirmed the RNA-seq data, although some of them missed statistical significance.

### MicroRNA-inhibition efficiency

**Figure 1:**
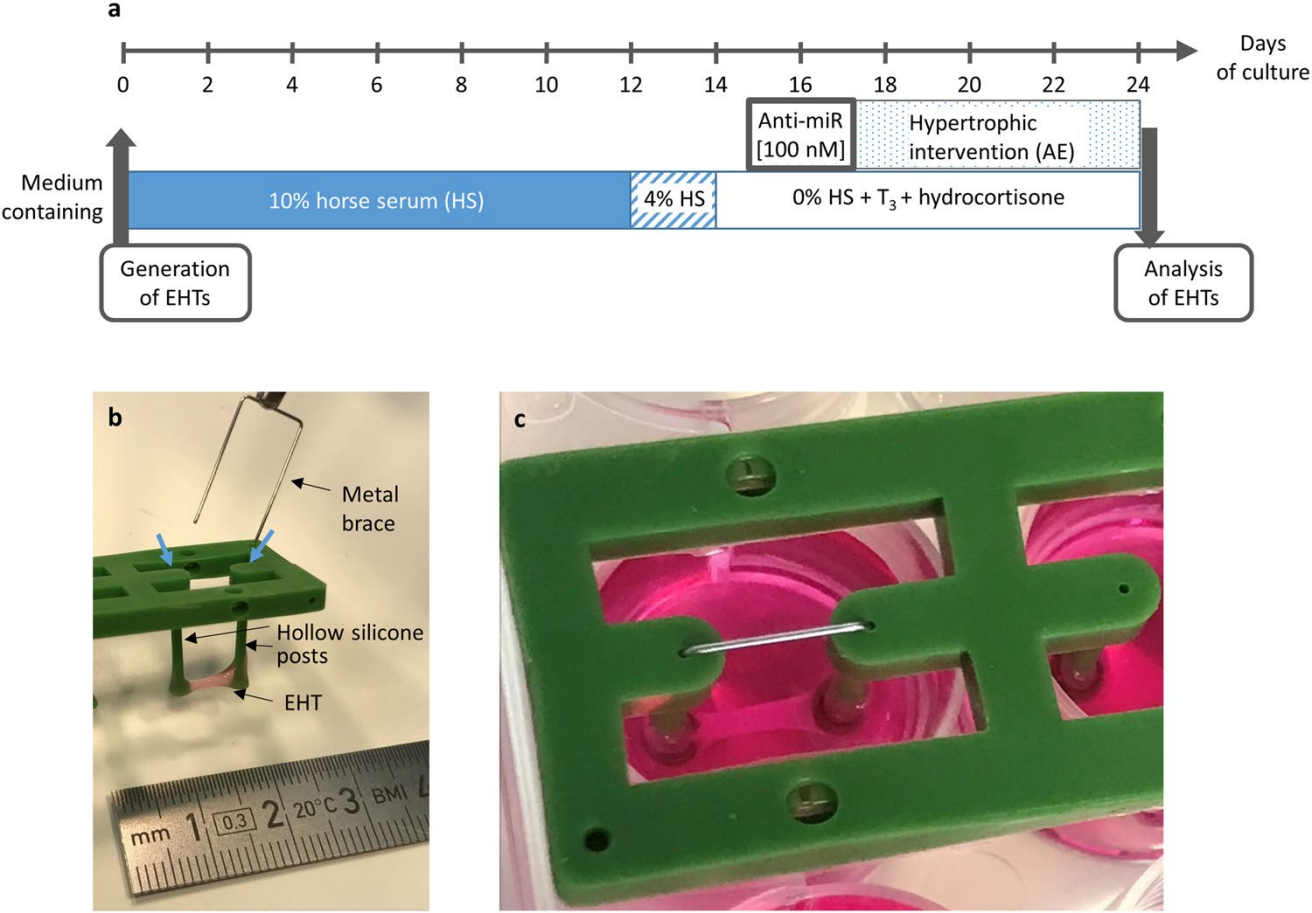
Engineered heart tissue (EHT) and the afterload enhancement (AE) procedure to induce hypertrophy. (**a**) Outline of the experimental procedure. (**b**) Photograph of an EHT taken out of the cell culture dish. To increase afterload of the EHTs, a metal brace is inserted into the opening of the posts (blue arrows). (**c**) An EHT in the cell culture dish during the AE-procedure.

**Figure 2:**
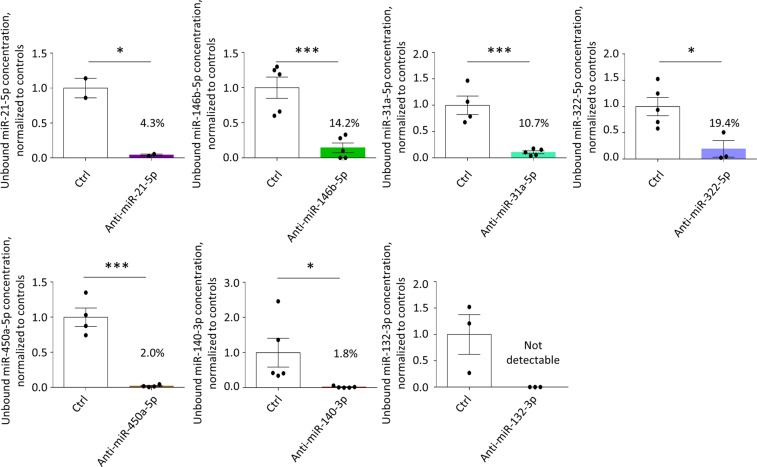
Concentrations of free microRNAs (i.e., not bound to an anti-miR) in EHTs treated with anti-miRs compared to control EHTs (Ctrl) at the end of the experiments, bars show mean ± SEM, n = 2–5 EHTs per group, Student’s unpaired t-test, *p < 0.05, ***p < 0.001.

The inhibition of the selected microRNAs was achieved by a simple singular transfection of LNA-anti-miRs under serum-free conditions over two days (day 15–17), prior to one week of AE (day 17–24; Fig. 1a). The latter was performed via insertion of stiff metal braces into the hollow silicone posts to which the EHTs were attached (Fig. 1b,c). All LNA-anti-miRs were between 16 and 20 nucleotides long, which is the typical length for *in vitro* experiments. The only exception was the shorter anti-miR-21-5p with only 15 nucleotides, which had been used for *in vivo* experiments before^20^. The sequences were chosen in a way that they always covered the seed  region and that they would also be suitable for the human orthologue of the targeted microRNAs (Table 1). All anti-miRs reduced the concentration of the targeted free microRNA to <20% of control on day 24 (Fig. 2), when EHTs were finally analyzed after the AE-procedure. The inhibition efficiency was higher for lowly abundant microRNAs (miR-450a-5p, miR-140-3p, miR-132-3p) than for highly abundant microRNAs (miR-21-5p, miR-146b-5p, miR-31a-5p, miR-322-5p).

### Attenuation of AE-induced contractile failure by anti-miR-140-3p-pretreatment

**Figure 3:**
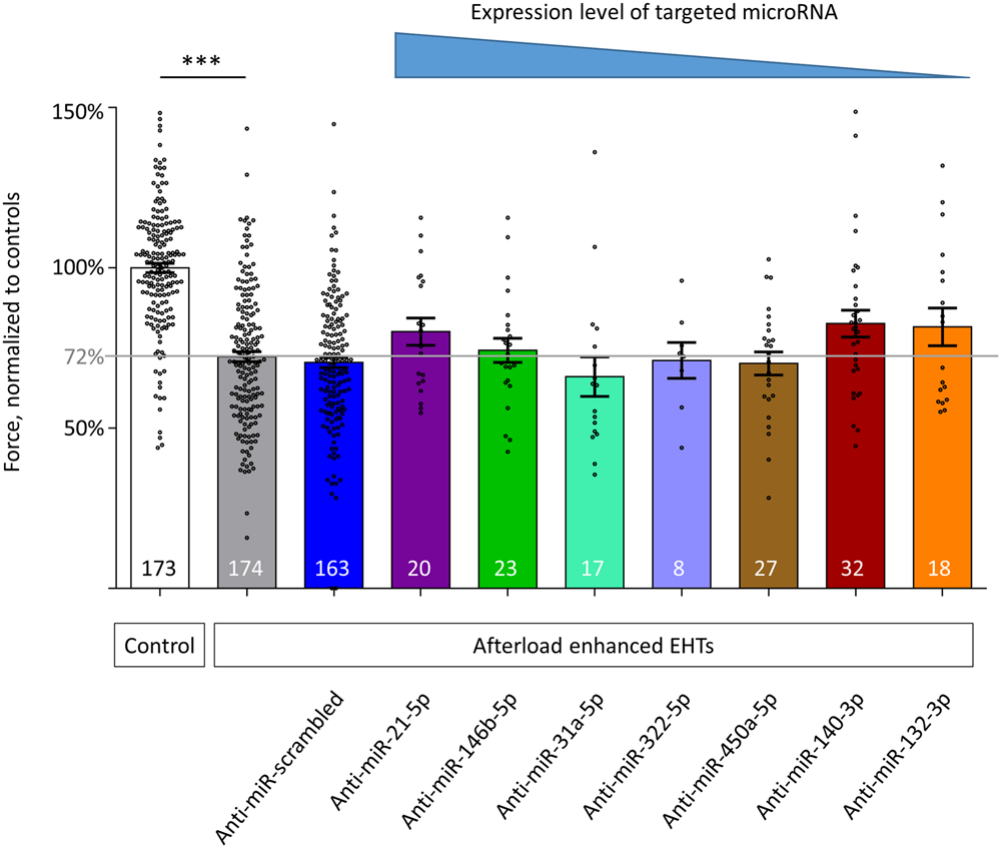
Contractile force of control EHTs, AE-EHTs, and AE-EHTs pretreated with anti-miRs. The grey line represents the average force of AE-EHTs in the absence of any anti-miR intervention, i.e. 72% of control EHTs. Bars show mean ± SEM, one-way ANOVA, Dunnett’s post-test for multiple comparisons against AE, ***p < 0.001, all numbers refer to total number of EHTs per group.

The pretreatment with LNA-anti-miRs in some cases tended to attenuate the contractile impairment of the one week of AE, which without pretreatment resulted in a 28% lower force. While – as expected from our previous results and the literature – anti-miR-21-5p and anti-miR-132-3p partially prevented this decline, no effect could be observed for anti-miR-146b-5p, anti-miR-322-5p and anti-miR-450a-5p, and we even observed an adverse effect of anti-miR-31a-5p. However, anti-miR-140-3p pretreatment was inclined to prevent the AE-induced contractile decline to a similar extent as anti-miR-21-5p and anti-miR-132-3p (Fig. 3).

### Shortening of anti-miR-140-3p to facilitate future *in vivo* applications

The LNA-anti-miR-140-3p we used in the small-range screen was 19 nucleotides long. Apart from one nucleotide both at the 5′-end and at the 3′-end, it covered the entire miR-140-3p sequence including the seed region. While this design leads to a reliable inhibition of target microRNAs when transfected *in vitro* (all microRNAs in Fig. 2 were targeted like that), its main disadvantage is that it cannot be taken up into cells unassisted. In contrast, slightly shorter (≤15 nucleotides) anti-miRs are well taken up by cells, rendering *in vivo* experiments possible^21^.

**Figure 4:**
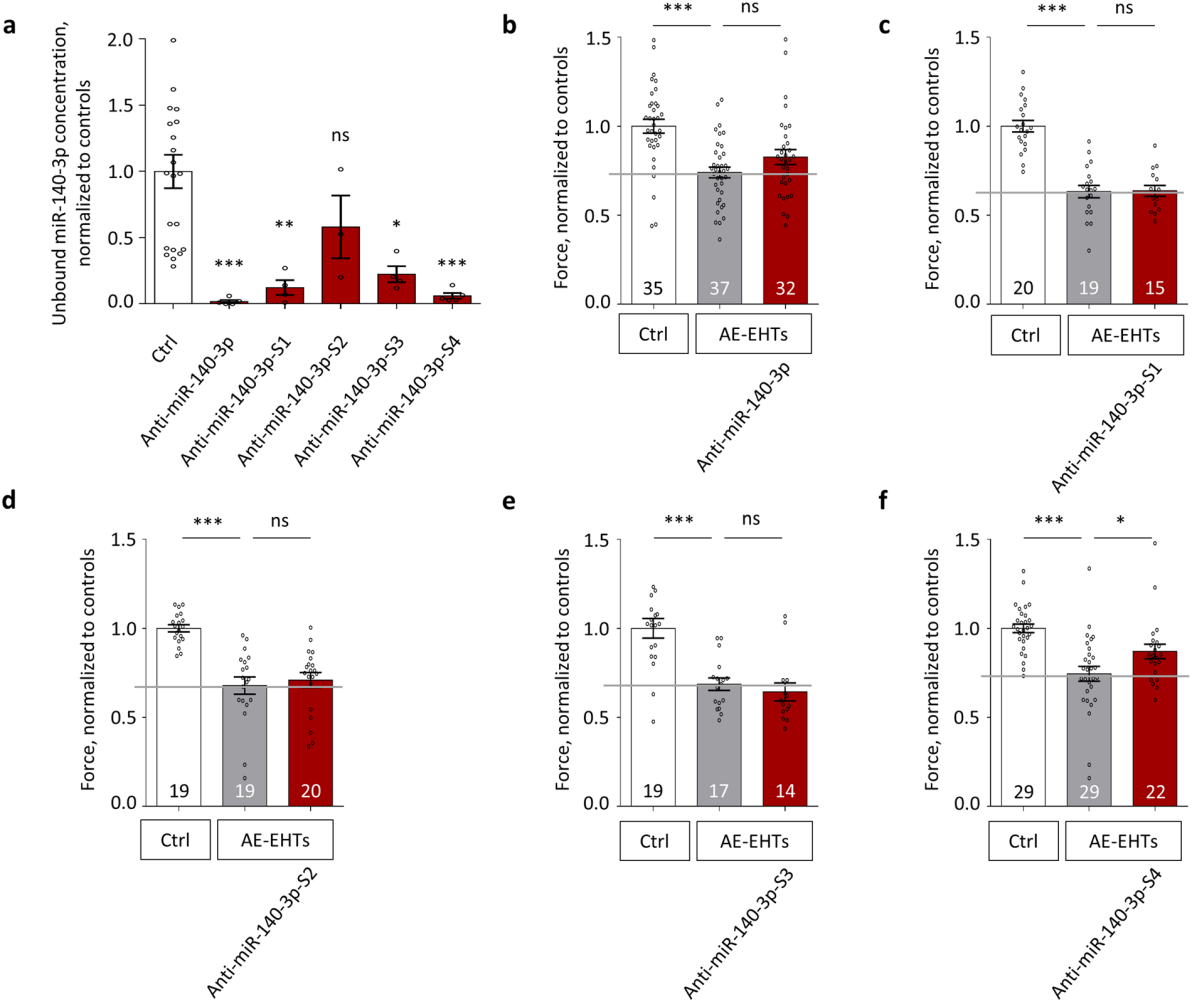
Evaluation of shorter anti-miRs targeting miR-140-3p. (**a**) Concentrations of free miR-140-3p (i.e., not bound to an LNA-anti-miR) at the end of the experiments, n = 3–22 EHTs per group. (**b**–**e**) Contractile force of Ctrl-EHTs, AE-EHTs and AE-EHTs pretreated with a long anti-miR-140-3p and four shorter anti-miR-140-3p (S1–S4). Bars show mean ± SEM, one-way ANOVA and Dunnett’s post-test for multiple comparisons against Ctrl, *p < 0.05, **p < 0.01, ***p < 0.001, all numbers refer to total number of EHTs per group.

We designed four shorter anti-miRs targeting miR-140-3p (S1–S4), each consisting of 14 or 15 nucleotides (Table 2). To counteract the decrease in binding affinity by shortening of the anti-miRs, we increased their relative LNA content from 26% to over 33%, which limited the drop in melting temperature to few degrees. Yet, the shorter anti-miRs S1-S3 were less efficient than the longer version, and only when two 5-carbon-methylated-cytosin-LNAs (5-Me-C-LNAs) were included (in anti-miR-140-3p-S4) an inhibition of miR-140-3p of >90% could be reached (Fig. 4a). All Anti-miR-140-3p-versions were tested with the same experimental procedure (Fig. 1a, i.e. transfection at 100 nM) as for the small-range screen. In contrast to the long anti-miR, which exerted a trend of a protective effect (Fig. 4b), the less effective shorter anti-miRs S1 (Fig. 4c), S2 (Fig. 4d) and S3 (Fig. 4e) did not protect against the AE-induced contractile failure. In contrast, the highly effective (94% inhibition) anti-miR-140-3p-S4 almost halved (−46%) the detrimental effect of AE (Fig. 4f). Taken data from all four inhibitors together, we found a significant negative correlation between unbound miR-140-3p concentration and contractile force of AE-EHTs (Fig. S3).

**Table 2:**
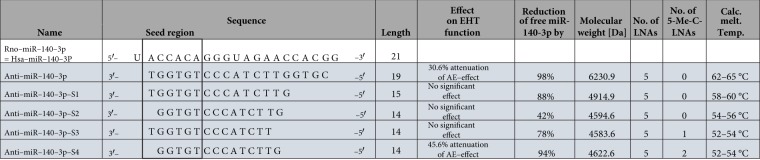
Molecular and functional details of miR-140-3p inhibitors.

### Further characterization of miR-140-3p inhibition in AE-EHTs

**Figure 5:**
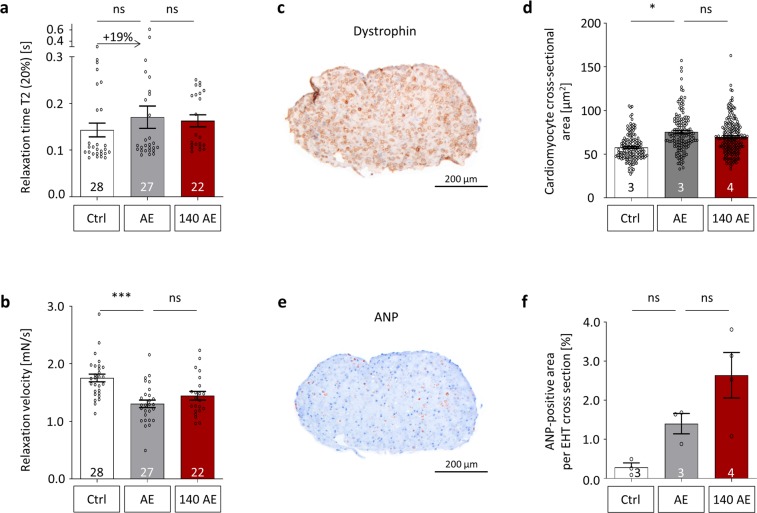
Further characterization of miR-140-3p inhibition with anti-miR-140-3p-S4 in AE-EHTs. Contractility analysis of (**a**) Relaxation time and (**b**) Relaxation velocity of Ctrl-EHTs, AE-EHTs and AE-EHTs pretreated with LNA-anti-miR-140-3p-S4 (140 AE) at the end of the experiments, n = 22–28 EHTs per group. Representative EHT cross sections stained for (**c**) Dystrophin and (**e**) ANP from which (**d**) Cardiomyocyte area and (**f**) ANP-positive area were quantified respectively, n = 3–4 EHTs per group; same groups as in (**a**,**b**). Bars show mean ± SEM, one-way ANOVA and Dunnett’s post-test for multiple comparisons against AE, *p < 0.05, ***p < 0.001.

The inhibitor anti-miR-140-3p-S4 was very effective and had a beneficial effect on contractile force, so we went on to further evaluate its effects on AE-EHTs. The changes of contraction kinetics due to AE included a tendency towards a prolonged relaxation time (+19%), which was less pronounced under miR-140 inhibition (Fig. 5a), as well as a significant reduction of relaxation velocity, which was partly prevented by anti-miR-140-3p pretreatment (Fig. 5b). As the hallmark of cardiac hypertrophy we also measured cardiomyocyte size from dystrophin-stained EHT cross sections (Fig. 5c) and found hypertrophied cells in AE-EHTs (76 µm^2^ on average) compared to control EHTs (58 µm^2^ on average). The mean cell size in anti-miR-140 pretreated EHTs was 70 µm^2^ and thereby in between the two other groups, yet not significantly different from AE-EHTs (Fig. 5d). Additionally we stained EHTs for atrial natriuretic peptide (ANP; Fig. 5e) and found a 5-fold increase in ANP-positive area per EHT cross section in AE-EHTs compared to controls (1.40% vs 0.28%) and an even larger area under miR-140 inhibition (2.6%; Fig. 5f). However, both groups did not differ statistically significantly from the control. Gene expression of other markers for pathological hypertrophy was analyzed using quantitative PCR, but no major effect of miR-140-3p inhibition could be observed (Fig. S4a).

To identify potential mRNA targets of miR-140-3p, we used a free online tool called miRDB^22^ and screened the top 40 candidates for transcriptional downregulation in AE-EHTs compared to controls utilizing a microarray expression analysis we published earlier^16^. Thereby we found two genes, namely Tab2 and Meis2, which could potentially be involved in miR-140-3p-related changes of EHT response to AE (Fig. S4b) and indeed gene expression analysis of one of the candidates, Tab2, revealed a tendency towards lower transcript levels in AE-EHTs compared to controls (fold change = 0.7) and a partial normalization under miR-140-3p inhibition (Fig. S4c).

## Discussion

In this study we explored functional consequences of inhibiting seven different microRNAs which have previously been shown to be upregulated in pathological cardiac hypertrophy induced by afterload enhancement in rat EHT. Using LNA-anti-miRs, we could achieve a powerful reduction of the free concentrations of all targeted microRNAs, which in the case of miR-21-5p, miR-132-3p and miR-140-3p attenuated afterload-induced contractile dysfunction. Chemical optimization of anti-miR-140-3p identified a short 14-mer LNA-anti-miR that effectively downregulated miR-140-3p in EHTs, when transfected at a concentration of 100 nM.

That it can quickly and easily discriminate between trends of detrimental and protective effects of miRNA-inhibition on contractility, represents one advantage of our functional testing strategy. While the inhibition of the miR-146b-5p, miR-322-5p (human orthologue miR-424-5p) or miR-450a-5p did not change the effect of AE on EHTs, the miR-31a-5p-inhibition (human orthologue miR-31-5p) even aggravated the contractile dysfunction.

We anticipated a protective tendency of the inhibition of the well-known miR-21-5p^18^ and miR-132-3p^19^ and thus designated them as positive controls. Although these microRNAs differ almost 1000-fold in the overall tissue concentration (miR-21-5p > miR-132-3p) and in their cell type distribution (miR-21-5p predominantly fibroblasts, miR-132-3p predominantly cardiomyocytes), these expectations have been met. This highlights another advantage of our small scale screening approach in EHTs over 2D-cardiomyocyte culture, as rat EHTs contain all native cardiac cell types (in addition to cardiomyocytes e.g. endothelial cells, fibroblasts, smooth muscle cells and immune cells) in which microRNAs or their inhibition could be beneficial. For a start, it is not even necessary to know the cell type expression pattern of targeted microRNAs. Furthermore, the non-cell-type specific microRNA inhibition approach in the heart will probably turn into a therapeutic option sooner than a more complex cell-type specific concept. Either way more research is needed to improve our mechanistic insights into miRNA regulation to progress translational implications. Studies using engineered tissues generated from human stem cell derived cardiomyocytes are helping to elucidate differences in miRNA-related effects between human and murine cells^23^ and are better suitable to investigate cell type-specific mechanisms, as these tissues are usually made of cardiomyocytes only^24^ or a defined ratio of cardiomyocytes and fibroblasts^25^. Another important aspect revolves around comparing anti-miR chemistries. Similar to our approach for miR-140-3p-inhibitors, Thum *et al*. tested multiple anti-miRs targeted against miR-21-5p to follow-up on conflicting study outcomes, pointing at varying efficacies of different inhibitors^26^.

In our initial screening experiment, the inhibition of miR-140-3p attenuated the contractile dysfunction and reveals miR-140-3p as a potential candidate for a novel drug target. The pre-miRNA-140 is of special interest as its 3p-strand had been misclassified as passenger strand both in humans and rodents, even though its expression is ~50-fold higher than its 5p-strand^27^. This misclassification presumably explains why miR-140-3p was not detected in earlier microRNA studies based on conventional arrays. After we had published this misclassification and the upregulation of miR-140-3p in our AE-EHT-system by RNA-Seq in 2015^17^, miR-140-3p has been frequently reported as a microRNA upregulated in diseased cardiac tissue, including a study that found miR-140-3p together with miR-132 and miR-210 to precisely predict cardiovascular death^28^. However, it was unknown whether miR-140-3p-upregulation was protective, detrimental or just collateral. To our knowledge, we are the first to test the functional consequences of miR-140-3p-inhibition on work performing cardiac tissue. In the last part of the project we shortened and modified our promising 19-mer inhibitor LNA-anti-miR-140-3p, so it should become more suitable for future animal experiments. The unassisted cellular uptake of antisense oligonucleotides was described and termed gymnosis by Stein *et al*.^21^. This process even works in terminally differentiated cells^29^, is most efficient with a phosphorothioate backbone (as in LNAs), but is minimal when using inhibitors longer than 16 nucleotides^21^. Thus, we aimed to use shorter inhibitors to simplify future *in vitro* experiments and - in particular – to create an inhibitor for *in vivo* applications.

We shortened the sequences mainly at the 5′-ends to keep the seed sequence largely preserved and increased the relative LNA-content to limit the drop in calculated melting temperature^30^. Nevertheless, our first short LNA-anti-miR-140-3p inhibitors (S1-S3) were less effective than the long version as evidenced by higher free miR-140-3p-concentrations in EHTs and by the lack of a protection against AE-induced contractile failure. Introduction of 5-carbon-methylated-cytosin-LNAs (5-Me-C-LNAs) resulted in an inhibitor (S4) that was highly potent, leading to a reduction of free miR-140-3p by 94%. The higher binding efficiency of 5-Me-C-LNA-containing oligonucleotides has been reported before and interestingly, the authors also reported the possibility of more complex interactions between oligonucleotides (in our case microRNA and LNA-anti-miR duplexes), namely triplexes or tetraplexes with melting temperatures above 85 °C^31^. This could be an explanation for the high binding affinity of S4, which might have a higher melting temperature than was calculated. Overall, our experience shows that calculation and prediction tools for short microRNA-inhibitors are still not perfect and a trial and error approach with subsequent experimental validation is unavoidable.

The potent inhibitor S4 in turn had the highest protective effect against AE. The decline in contractile force was halved at a concentration of 100 nM, similar to what we had observed with the LNA-anti-miR-21-5p^17^. The correlation between strong inhibition of miR-140-3p and favorable functional consequence (Table 2 and Fig. S3) points to a possible causal relationship.

In line with the beneficial effect on contractile force, we found other aspects of pathological hypertrophy to be slightly attenuated by miR-140-3p inhibition, including relaxation velocity and cardiomyocyte size. On the other hand, ANP expression was more pronounced after anti-miR-140-3p pretreatment than in AE-EHTs, which could again hint at a favorable effect, as natriuretic peptides like ANP and BNP have not only been shown to be cardiac biomarkers^32^, but also to have cardioprotective functions^9,33^. One of which is inhibiting cardiomyocyte hypertrophy via inhibition of mitogen-activated protein kinase (MAPK) signaling^34^ and this mode of action on cellular level is likely to play an important role in the AE-EHT, as circulatory functions of natriuretic peptides can be excluded in this *in vitro* model.

Interestingly, a potential miR-140-3p target in AE-EHTs is TGF-Beta Activated Kinase 1 (TAK1/MAP3K7) Binding Protein 2 (TAB2), which forms a complex with TAB1 (TAK1-binding protein 1) and TAK1 that activates MAPK signaling and the latter has also been shown to provoke cardiac hypertrophy *in vivo*^35^. In addition Liu and colleagues^36^ have found that TAB2 is a direct binding partner of RCAN1 (regulator of calcineurin 1), indicating a regulatory role of the TAK1-TAB1-TAB2 complex between MAPK and calcineurin signaling pathways and highlighting the complexity of hypertrophic responses.

When we analyzed TAB2 expression in EHTs, we found the expected trend (downregulation under AE conditions compared to controls and slightly higher expression in AE-EHTs after miR-140-3p inhibition), but changes were not statistically significant. Generally microRNAs are thought to be components of vast regulatory networks with usually rather moderate effect on target mRNA and additionally, multiple microRNAs can target the same gene. Thus uncovering mechanistic links represents a demanding systems biology task^37^. However, if the beneficial miR-140-3p-inhibition holds true in animal experiments this endeavor should be undertaken.

A limitation of our study is that we did not investigate which of the different cell types in EHTs are involved and/or targeted and how exactly the inhibition of miR-140-3p leads to an attenuation of the contractile decline caused by AE. Instead we rather focused on an advantage of the EHT-system, namely the possibility to easily measure contractile force, which can be regarded as composite endpoint of all molecular changes occurring. Besides the anticipated protective effect of the miR-21-5p and miR-132-3p-inhibition we analyzed the inhibition of other miRs which were neutral or detrimental, but also the inhibition miR-140-3p which turned out to be protective against the detrimental effect of afterload increase.

## Methods

### Generation of engineered heart tissue (EHT)

The generation of rat EHTs is standardized and was comprehensively described previously^16^. Shortly, ventricular rat heart cells were obtained by a trypsin/DNase digestion of ventricles from neonatal Wistar rats (postnatal day 0 to 3). For each EHT, 500,000 cells were mixed with fibrinogen and thrombin at a final volume of 100 µl and then quickly pipetted into casting molds, into which two hollow silicone posts protruded. After 2 hours, the fibrin block containing the heart cells could be taken out of the molds, attached to the two silicone posts, in between which it was spanned (Fig. 1a) and transferred to a new 24-well cell culture plate. Subsequently, these EHTs were cultured in medium on a DMEM-basis supplemented with 10% inactivated horse serum, penicillin/streptomycin, insulin and aprotinin to prevent rapid fibrin degradation.

### Application of miRNA-inhibitors

After 12 days, the horse serum concentration in the EHT-medium was decreased to 4%, after 14 days and for the rest of the experiments, horse serum was completely omitted. The medium was instead supplemented with low concentrations of triiodothyronine (T_3_, 0.5 ng/ml) and hydrocortisone (50 ng/ml). Antagomirs were delivered via simple addition to the culture medium at a concentration of 80 µg/ml for three days (day 18–21). LNA-anti-miRs were transfected by a cationic polymer mediated endocytosis (TurboFect, ThermoFisher) at a concentration of 100 nM for three days (day 18–21) in the initial comparison with antagomirs (Fig. S1) and for two days (day 15–17) in all other experiments. We validated the procedure by transfecting EHTs with a fluorescently labeled LNA-5′-6-FAM-anti-miR with a scrambled sequence for 2 days and fixed EHTs one week after transfection overnight in Roti-Histofix 4%. Whole mount EHTs were then imaged on a Zeiss LSM800 confocal microscope (Fig. S2). All LNA-anti-miRs were obtained from Exiqon (sequences in Table 1). The melting temperatures were calculated with the tools developed by Owczarzy *et al*.^30^.

### Induction of hypertrophy and contractility analysis of EHTs

On day 17, EHTs were subjected to an increase in afterload by inserting stiff metal braces into the hollow silicone posts that hold the EHTs (Fig. 1b,c). A 7 day-intervention (Fig. 1a) leads to hypertrophy, a decrease in force and many other aspects of pathological cardiac hypertrophy^16^. The contractility of EHTs was measured every other day by automated sterile video recordings and analysis using a custom built measurement system (EHT Technologies) and pattern recognition software (CTMV).

### RNA isolation and quantification

EHTs were homogenized with steel beads in a highly chaotropic isolation buffer. A phenol-chloroform based extraction was performed and from the aqueous phase, large RNA (mRNA) and small RNA species (microRNA) were extracted separately with the help of isopropanol and silica columns (Lexogen Split RNA Extraction Kit). Complementary DNA of microRNAs was produced by first adding a poly-A tail and then by performing a reverse transcription reaction using a poly-T primer with a 3′-degenerate anchor and a 5′-universal tag. The reverse transcription was performed at 42 °C, well below the melting temperature of a microRNA/LNA-anti-miR-duplex. Thus, only active (unbound) microRNAs could be reversely transcribed. For the subsequent quantitative PCR, commercially available microRNA-specific LNA-enhanced primers were employed (Exiqon). The microRNAs 181-5p, 486-5p and 143-3p served as internal controls. For reverse transcription of mRNA and the subsequent qPCR, the High Capacity cDNA Reverse Transcription Kit (Applied Biosystems) and HOT FIREPol EvaGreen qPCR Mix Plus (Solis BioDyne) were used according to manufacturers’ instructions. Beta-glucuronidase (Gusb) served as internal control gene to countervail variability; mRNA primer sequences can all be found in the supplementary material (Table. S1). All qPCR reactions were performed on an ABI PRISM 7900HT Sequence detection system (Applied Biosystems) and data analyses were carried out using the ΔΔ^−Ct^ method.

### Histology

For immunohistochemistry, EHTs were fixed with formaldehyde overnight and embedded in paraffin. 3 µm thick sections were cut at the center of the tissue and stained using mouse monoclonal antibodies targeted either against dystrophin (Millipore, MAB1645) at a dilution of 1:200 with 60 min antigen retrieval in EDTA-buffer, pH 8.0 or against ANP (Santa Cruz, sc-80686) at a dilution of 1:500 with 60 min antigen retrieval in citrate-buffer, pH 6.0. Histological sections were visualized with the UltraView Universal DAB Detection Kit (Roche) and microscopic images were taken with either a Hamamatsu NanoZoomer 2.0-HT slide scanner or an Axioskop 2 microscope (Zeiss). Average cardiomyocyte cross-sectional areas were determined in a blinded manner from dystrophin-positive cells by manually measuring 50 random cells per tissue section in ImageJ, using the freehand trace function. To determine the percentage of ANP-positive area an RGB color threshold was applied to the images (R251, G165, B147) and the area was normalized to the cross-sectional area of the EHT.

### Statistics

Results are presented as bars showing mean ± SEM and individual values. All statistical tests were performed in GraphPad Prism version 8.0.2. In detail, 1-way ANOVA and Dunnett’s or Sidak’s multiple comparison post-test (to compare to controls) was used for more than 2 groups, or Student’s unpaired t-test for 2 groups. P < 0.05 or less was considered statistically significant. P-values are displayed graphically as follows: *p < 0.05, **p < 0.01, ***p < 0.001, ns = not significant.

### Ethical standards

All animal work was approved by the local Animal Welfare Committee of the City of Hamburg, Germany (approval #08/14) and was conducted in accordance with the Guide for the Care and Use of Laboratory Animals as adopted by the United States National Institutes of Health (NIH publication No. 85-23, revised 1996). The manuscript does not contain clinical studies or patient data.

## Supplementary information

Supplementary information

## Data Availability

All data generated or analyzed during this study are included in this published article and its Supplementary Information files.
