# Supplementary material for: Blockade of miR-140-3p prevents functional deterioration in afterload-enhanced engineered heart tissue
Source: Sci Rep. 2019 Aug 7;9:11494. doi: 10.1038/s41598-019-46818-0 (PMC6686025; doi:10.1038/s41598-019-46818-0)
Supplement: Supplementary file 1 — Supplementary information [file 41598_2019_46818_MOESM1_ESM.pdf]

Blockade of miR-140-3p prevents functional deterioration in afterload-enhanced engineered heart tissue

Journal:

Scientific Reports

Authors:

Tessa R. Werner[1,2], Ann-Cathrin Kunze[1,2], Justus Stenzig[1,2] , Thomas Eschenhagen[1,2], Marc N. Hirt[1,2]

Affiliations:

[1]Department of Experimental Pharmacology and Toxicology, University Medical Center Hamburg-Eppendorf, Hamburg, Germany; [2]DZHK (German Centre for Cardiovascular Research), partner site Hamburg/Kiel/Lübeck, Germany

Corresponding author:

Marc N. Hirt, E-mail: m.hirt@uke.de

Fig. S1

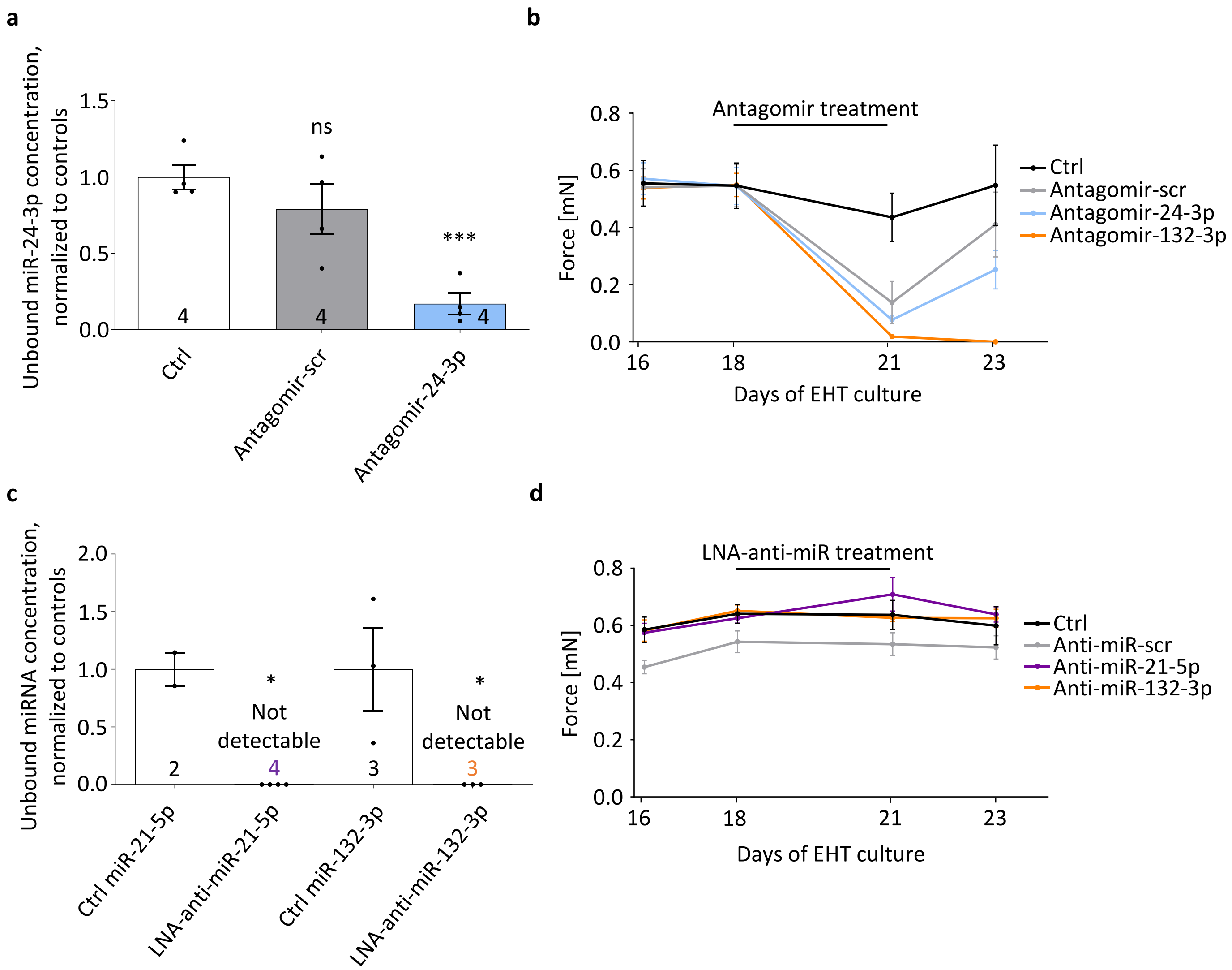

Fig. S1 Comparison of anti-miR chemistries shows acute toxicity of antagomirs. a) and c) Concentrations of free miRNAs (i.e., not bound to an inhibitor) at the end of the experiment (day 28), a) n=4 EHTs per group, bars show mean±SEM, one-way ANOVA and Dunnett's post-test for multiple comparisons against Ctrl, ***p<0.001; c) n=2-4 EHTs per group, bars show mean±SEM, one-way ANOVA and Sidak's post-test for multiple comparisons against corresponding Ctrl, *p<0.05. b) and d) Contractile force over time of control EHTs and EHTs treated with b) 80 µg/ml of a scrambled sequence antagomir or targeted against miR-132-3p or miR-24-3p for three days, n=8 EHTs from d16-d21 and n=4 on day 23 per group or d) 100nM of a scrambled sequence LNA-anti-miR or targeted against miR-132-3p or miR-21-5p for three days, n=6 EHTs.

Fig. S2

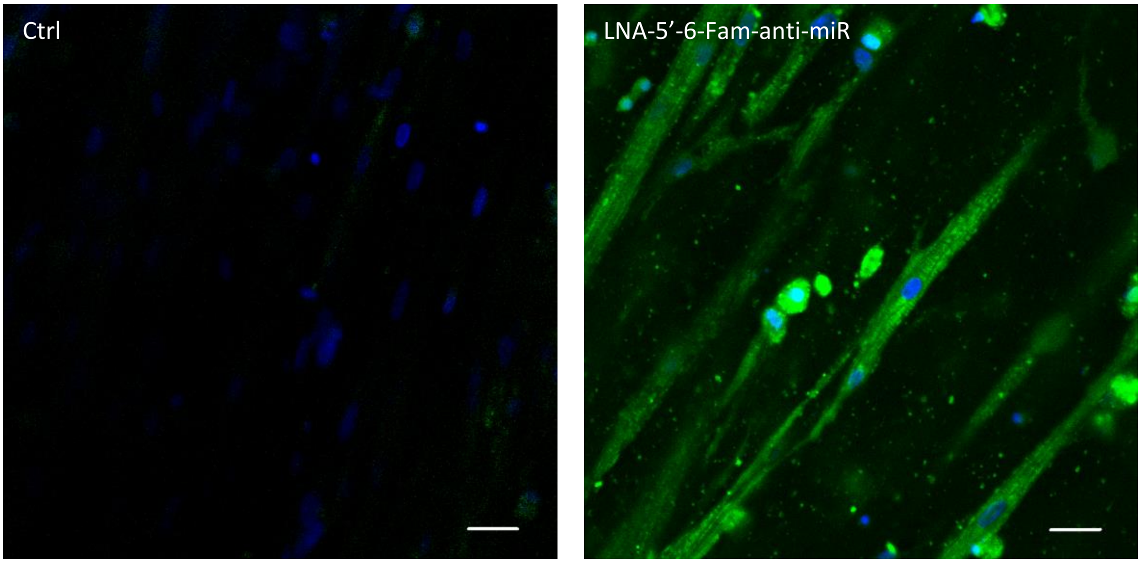

Fig. S2 Immunofluorescence of an untreated control EHT (Ctrl) and after transfection with 100 nM fluorescently labeled LNA-5'-6-Fam-anti-miR for 2 days showing the anti-miR penetrating cells throughout the EHT. Nuclei were stained using DAPI, scale bar = 20 µm.

Fig. S3

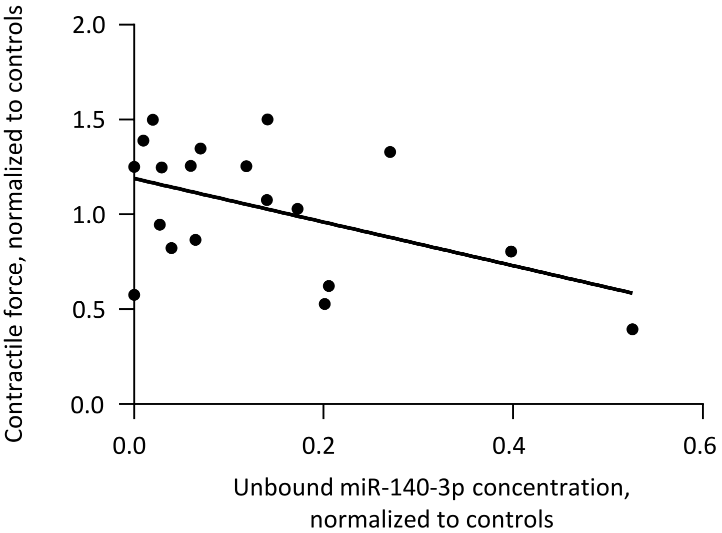

Fig. S3 Correlation of contractile force and unbound miR-140-3p in EHTs after transfection with LNA-anti-miRs targeted against miR-140-3p and one week of afterload enhancement. The results of the Pearson correlation are as follows: $r = -0.48$, $r^2 = 0.23$ and $p = 0.0393$, showing a significant negative correlation between the two variables.

Fig. S4

a

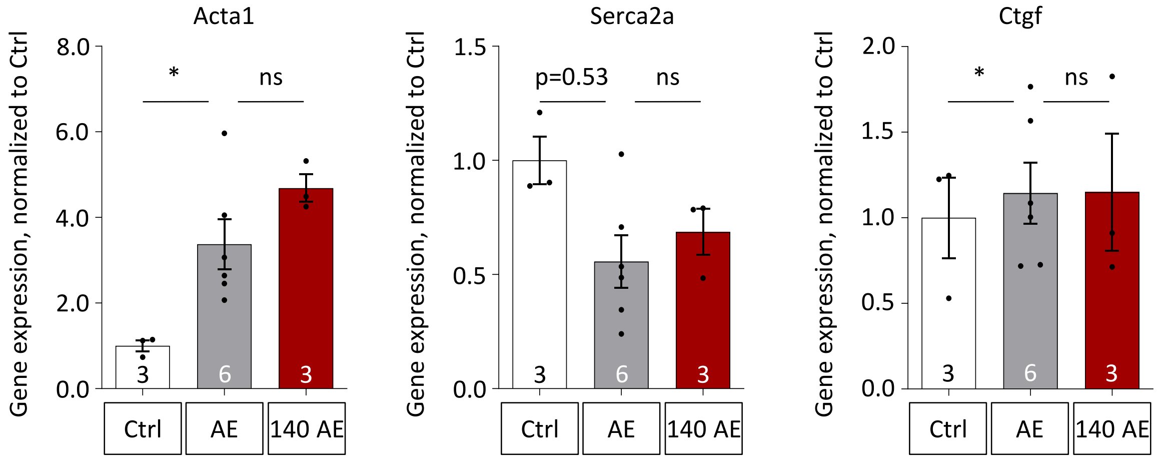

b

Gene	miRDB		Microarray expression analysis	
	Target rank	Target score (max = 100)	FC (AE/Ctrl)	p-value
Tab2	2	96	0.65	<0.00001
Meis2	37	85	0.55	0.00002

c

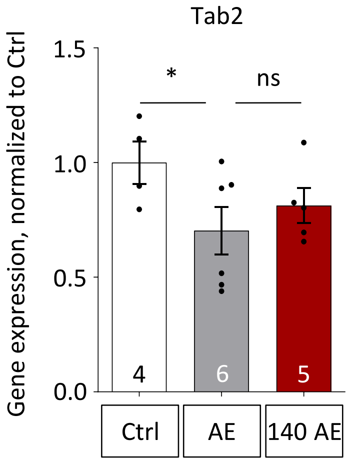

Fig. S4 Gene expression analysis of hypertrophic markers and miR-140 target identification. a) qPCR analysis of Acta1, Serca2a and Ctgf in Ctrl-EHTs, AE-EHTs and AE-EHTs with anti-miR-140-3p pretreatment, b) miR-140 target identification using miRDB and microarray expression analysis in AE-EHTs vs controls, revealing Tab2 as most likely miR-140-target in AE-EHTs, FC=fold change. c) qPCR analysis of Tab2 in Ctrl, AE- and anti-miR-140 pretreated EHTs. a) and c) n=3-6 EHTs per group, bars show mean±SEM, one-way ANOVA and Dunnett's post-test for multiple comparisons against AE.

Tab. S1

Tab. S1 Primer sequences for mRNA qPCR analysis

Gene symbol	NM_number	Forward primer	Reverse primer
Gusb	NM_017015.1	CCCGCATGTCCCCAAGACGG	CGGCACGGAAGCTCCACAGG
Acta1	NM_019212.2	AGGACCTGTACGCCAACAAC	ACATCTGCTGGAAGGTGGAC
Atp2a2	NM_001110139.2	GGCTCGTGGGCTCCATCTGC	TCCAGTATTGCAGGCTCCAGGT
Ctgf	NM_022266.2	GCGAGCCAACTGCCTGGTCC	GCGTCCGGATGCACTTTTTGCC
Tab2	NM_001012062.1	TGGGATCTGATGATGCTGCC	CTTCGAGTGAAGGTATCTGGGA
